# Using magnetic resonance microscopy to study the growth dynamics of a glioma spheroid in collagen I: A case study

**DOI:** 10.1186/1471-2342-8-3

**Published:** 2008-01-29

**Authors:** Shuning Huang, David Vader, Zhihui Wang, Anat Stemmer-Rachamimov, David A Weitz, Guangping Dai, Bruce R Rosen, Thomas S Deisboeck

**Affiliations:** 1 Harvard-MIT (HST) Athinoula A. Martinos Center for Biomedical Imaging, Massachusetts General Hospital, Charlestown, MA 02129, USA; 2 Harvard-MIT (HST) Massachusetts Institute of Technology, Cambridge, MA 02139, USA; 3 School of Engineering and Applied Sciences (SEAS), Harvard University, Cambridge, MA 02138, USA; 4 Department of Pathology (Neuropathology), Massachusetts General Hospital, Boston, MA 02114, USA

## Abstract

**Background:**

Highly malignant gliomas are characterized by rapid growth, extensive local tissue infiltration and the resulting overall dismal clinical outcome. Gaining any additional insights into the complex interaction between this aggressive brain tumor and its microenvironment is therefore critical. Currently, the standard imaging modalities to investigate the crucial interface between tumor growth and invasion *in vitro *are light and confocal laser scanning microscopy. While immensely useful in cell culture, integrating these modalities with this cancer's clinical imaging method of choice, i.e. MRI, is a non-trivial endeavour. However, this integration is necessary, should advanced computational modeling be able to utilize these *in vitro *data to eventually predict growth behaviour *in vivo*. We therefore argue that employing the *same *imaging modality for both the experimental setting *and *the clinical situation it represents should have significant value from a data integration perspective. In this case study, we have investigated the feasibility of using a specific form of MRI, i.e. magnetic resonance microscopy or MRM, to study the expansion dynamics of a multicellular tumor spheroid in a collagen type I gel.

**Methods:**

An U87mEGFR human giloblastoma multicellular spheroid (MTS) containing approximately 4·10^3 ^cells was generated and pipetted into a collagen I gel. The sample was then imaged using a T_2_-weighted 3D spoiled gradient echo pulse sequence on a 14T MRI scanner over a period of 12 hours with a temporal resolution of 3 hours at room temperature. Standard histopathology was performed on the MRM sample, as well as on control samples.

**Results:**

We were able to acquire three-dimensional MR images with a spatial resolution of 24 × 24 × 24 μm^3^. Our MRM data successfully documented the volumetric growth dynamics of an MTS in a collagen I gel over the 12-hour period. The histopathology results confirmed cell viability in the MRM sample, yet displayed distinct patterns of cell proliferation and invasion as compared to control.

**Conclusion:**

In this study, we demonstrate that a specific form of MRI, i.e. magnetic resonance microscopy or MRM, *can *be used to study the dynamic growth of a multicellular tumor spheroid (MTS) with a single cell scale spatial resolution that approaches the level of light microscopy. We argue that MRM can be employed as a complementary non-invasive tool to characterize microscopic MTS expansion, and thus, together with integrative computational modeling, may allow bridging of the experimental and clinical scales more readily.

## Background

High-grade malignant gliomas are characterized by rapid volumetric growth and extensive local tissue infiltration. Despite all efforts to improve diagnostics and therapy, the outcome remains dismal with a five-year survival rate below 3.3% in the main age group of 45-years and older [1]. Since the surrounding tissue is thought to impact, perhaps even guide the tumor's invasive patterns, much weight is currently being put on better understanding the dynamic interaction of an expanding brain tumor with its microenvironment. As such, following an interdisciplinary approach, we have previously employed an *in vitro *glioma multicellular tumor spheroid (MTS) model to investigate the spatial and temporal dynamics of MTS expansion within extracellular matrix environments [2-4], and started to model them *in silico *[5-7]. In these studies, various aspects of the interaction between a growing spheroid system and its environment were studied and mathematically modeled, including extracellular matrix concentration, mechanical forces, and invasion directionality.

In the 1980s, several groups independently acquired microscopic images of different biological samples using magnetic resonance spectroscopy [8-10]. These early studies demonstrated the feasibility of magnetic resonance microscopic imaging with its unique sensitivity to tissue water environment. Since then, with continued advances in magnetic resonance imaging (MRI) techniques (incl. stronger gradient coils, improved RF coil design, and more powerful computers), the imaging resolution has steadily increased. We are now able to acquire three-dimensional (3D) images at a high signal to noise ratio with a resolution approaching the single cell level. This allows us to employ MRI, the imaging modality of choice *in vivo*, to study these microscopic tumor models also *in vitro*.

We have previously reported that magnetic resonance microscopy (MRM) enables us to distinguish an MTS of roughly 250 μm in diameter in a collagen I gel and provides us with a true 3D view of the MTS within the matrix [11]. However, the time required to obtain high-resolution 3D images was considerably long, typically 10 hours. To dynamically monitor MTS growth, we would have to increase the temporal resolution while maintaining a comparable signal to noise ratio (SNR). For this very purpose, we applied Gd-DTPA, the contrast agent widely used in clinics, to the collagen I matrix, achieving not only a significantly shortened MR imaging time down to 3 hours, but also increasing the SNR from 20 to over 40. Therefore, we are able to dynamically monitor both global and local changes of the MTS over a 12-hour period at a temporal resolution of 3 hours and an isotropic spatial resolution of 24 μm. Using conventional immunohistochemistry techniques we were able to confirm cell viability in the MRM sample post imaging, as well as to find patterns of cell proliferation and invasion that seem distinctively different from the controls and thus warrant further analyses.

In summary, we argue that MRM enables us to examine MTS growth dynamically with a true 3D view. The nature of MRM data allows us to segment the spheroid's contour out of its microenvironment, reconstruct and visualize its surface, and subsequently analyze the MTS' distinct 3D dynamics from any selected angle. Tracking tumor expansion non-invasively down to the 'single-cell' scale by using a clinically relevant imaging modality should facilitate data integration, and, in combination with *in silico *modeling, will yield valuable insights into the critical interaction between the tumor and its microenvironment.

## Methods

### Multicellular tumor spheroid (MTS) and extracellular matrix

#### Cell culture

The U87mEGFR [11-13] cell line is cultured in 10 mL of high-glucose Dulbecco's Modified Essential Medium (DMEM; Invitrogen, Carlsbad, CA), supplemented with 1% Penicillin-Streptomycin (PS; Invitrogen), 10% Fetal Bovine Serum (FBS; JRH Biosciences, Lenexa, KS), 0.5 mg/mL Geneticin (Invitrogen) and 20 mM hepes buffer (Invitrogen) in 10 cm diameter Petri dishes (Corning, Corning, NY). Once cells are confluent, they are twice rinsed with Phosphate Buffered Saline (Invitrogen) and detach after the addition of 1 mL Trypsin-EDTA (Invitrogen). After 5–10 min, 9 mL of fresh cell media is added to neutralize the Trypsin. The cell solution is then transferred into a 15 mL centrifuge tube and centrifuged for 5 min at 1200 RPM (~2·10^3 ^m/s^2^). After aspirating the supernatant, the cells are resuspended in fresh media at a concentration of 2·10^5^/mL.

#### Multicellular spheroids

Spheroids are then generated using the hanging droplet method [14], with a drop size of 20 μL, which seeds ~4·10^3 ^cells in each spheroid to yield a diameter of approximately 400 μm. Briefly, multiple droplets of 20 μL of cell solution are pipetted onto the inside of a 100 mm-diameter Petri dish cover. After placing the cover back on a culture medium-filled dish, the dish itself is placed in the incubator (5% CO_2_, 37°C). Surface tension maintains droplet integrity, while gravity pulls cells together at the bottom of each droplet. Cells are left to form spheroids in the incubator for 3 days before they are collected.

#### Extracellular matrix

The extracellular matrix (ECM) model is a 1.5 mg/mL bovine collagen type I (Inamed Biomaterials, Fremont, CA) matrix supplemented with 10% FBS, 10% 10X Minimum Essential Medium (MEM; Invitrogen), 1% PS, 50 mM sodium bicarbonate (NaHCO_3_; Sigma, St. Louis, MO) buffer. For image enhancement, 10 mM Gd-DTPA (Magnevist; Berlex, Wayne, NJ, U.S.A.) is added to the collagen solution. To induce polymerization, a few μL of 1 M sodium hydroxide (NaOH; Sigma) are added until a neutral pH is reached. The choice of 1.5 mg/mL concentration used here is based on work by Kaufman et al. [2] and represents a reasonable compromise between achieving a sufficiently high viscoelastic modulus while avoiding blocking cell motility altogether.

#### Sample preparation

For each sample, we pipette 280 μLs of collagen solution into a μPCR tube (VWR, West Chester, PA). After 15–30 min in the incubator to allow the gel to initiate polymerization, a spheroid is pipetted into this collagen gel, which is then placed back in the incubator for several hours to allow cells to attach properly to the ECM before imaging. To assess the impact of MRM on cell viability, we compared the histology of the MR-imaged specimen with a control group that was treated identically but for one difference: instead of being imaged overnight, it was kept in the incubator. As soon as the experiment was halted, both the imaged specimen and the control samples were fixed, sectioned and stained.

### Magnetic resonance microscopy

We first measured Gd-DTPA (Magnevist) relaxivity in water at 14T (Magnex, 89 mm vertical bore, gradient strength 100 gauss/cm, Bruker Biospin System) using the conventional inversion recovery spin echo sequence at three different concentrations (0.075, 2.5, 5 mM). The Gd-DTPA concentration (10 mM) used here was then determined based on its dosage (0.2 – 0.5 mmol/kg) in MRI mouse model studies [15,16] and its relaxivity at 14T.

The MTS in collagen with 10 mM Gd-DTPA was imaged using MRM at 14T. Specifically, we first used a modified FLASH pulse sequence to acquire multi-slice multi-echo (TR/TE = 400/3 8 13 18 ms; FOV = 0.95 cm, 256 × 256; slice thickness = 400 μm; α = 30) images with an in-plane resolution of 37 × 37 μm^2 ^to localize the MTS in the collagen I gel. A quick T_1 _measurement was then performed using a modified IR_RARE sequence (TR/TE: 1500/7.86 ms; rare factor: 2; matrix size: 128 × 128; 10 slices with 0.4 mm slice thickness; FOV: 0.6 cm; TI: 5.621 55.620 105.620 205.620 505.620 1005.620 2005.620 ms). A three-dimension spoiled gradient echo (FLASH) sequence (TR/TE = 20/5.5 ms; FOV = 1.2 × 0.6 × 0.6; matrix size: 512 × 256 × 256; signal was optimized at the Ernst angle) was then used to acquire high-resolution (24 μm isotropic) microscopic images every three hours for a total of 12 hours. After imaging, the sample was fixed for histopathological analysis as described below. MR images were displayed employing an ImageJ software package [17] and then segmented using the 3D Slicer software package [18]. T_1 _of the sample and Gd-DTPA relaxivity were fitted using Matlab (Mathwork, Inc., Natick, MA). After the MTS was manually segmented for each time point, its corresponding volume and surface area were calculated.

### Histology

The spheroids were harvested and processed as described before [4], fixed in 10% formalin and embedded in paraffin. Blocks were serially sectioned in 7 μm thick sections and stained with hematoxylin and eosin (H&E). In order to assess the proliferative activity, immunohistochemistry was performed by using cell cycle-unspecific MIB1 antibody (which detects the nuclear Ki-67 antibody so that quiescent cells (G0) remain unstained) (DAKO, M7240, USA; 1:50 dilution). The so called MIB-1 labeling index was then calculated as the fraction of MIB-1 positive cells from the total number of cells. In addition, images of the test specimen and control were printed. The number of invading cells was counted and the distance of each cell was calculated from a circle drawn at the circumference of the spheroid. The distances were measured by drawing the shortest straight line from the circle around the spheroid to the invading cells. The measurements are in cm, but do not represent true distance of invasion (due to magnification of the image printed), but rather represent an [arbitrary unit] by which distances of the cells from the two MTS, i.e. 14T specimen versus control, can be compared.

## Results

### Magnetic resonance microscopy of MTS in collagen I gel

Prior to imaging the MTS, we characterized the r_1 _relaxivity (longitudinal relaxivity) of Gd-DTPA at 14T and the SNR improvement after adding 10 mM Gd-DTPA into the collagen I gel that did not contain any tumor cells. Table 1 shows that with 10 mM Gd-DTPA, we were able to reduce the T_1 _relaxation time of the collagen gel from around 3000 ms to about 24 ms. The imaging time was reduced from 10 hours to 3 hours with a two-fold increase of SNR (from 20 to over 40).

**Table 1:** MRI properties of Gd-DTPA and collagen I gel.

	**Gd-DTPA**	**Collagen I Gel without 10 mM Gd**	**Collagen I Gel with 10 mM Gd**
**r1 (1 mM^-1 ^sec^-1^)**	3.88 ± 0.12		
**R_1 _(sec^-1^)**		0.33	42.32
**SNR**		16	42

Measured longitudinal relaxivity of Gd-DTPA at 14 T, corresponding R_1 _changes of collagen I gel after adding 10 mM Gd-DTPA, and the resulting SNR improvement.

**Figure 1:**
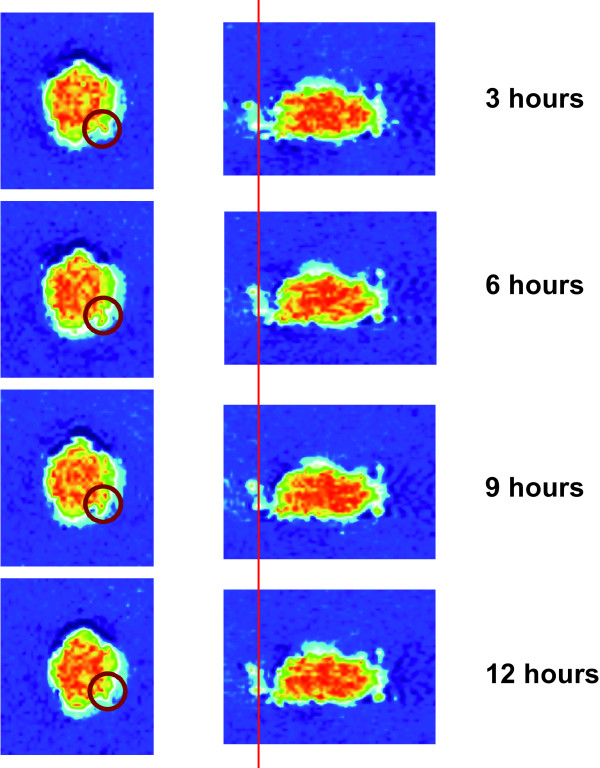
**MRM images that show the MTS growth over time from different angles**. The images to the left depict the *axial *view, whereas those to the right show an arbitrary angle. These 2D images confirm the surface heterogeneity that emerges already 12 hrs post placement of the MTS into the gel, and depict a small group of invasive cells (images on the right) that can also be seen in **Figures 2(A) **and **3**. On the other hand, images on the left demonstrate that certain parts of the solid spheroid actually grew into the gel (*red circle*).

The original MRM images of the MTS specimen are shown in Figure 1. The time series clearly documents the growth of the spheroid from different angles and at different planes. Even with the 2D view, MRM showed different patterns of cellular growth. Images on the left in Figure 1 show cells growing attached to the main body (red circle); while those on the right show some cells seemingly moving away from the main spheroid. It becomes apparent that with reduced imaging time and increased SNR, MRM can indeed monitor the spatio-temporal expansion of an MTS system.

### Segmentation and 3D visualization of MTS

**Figure 2:**
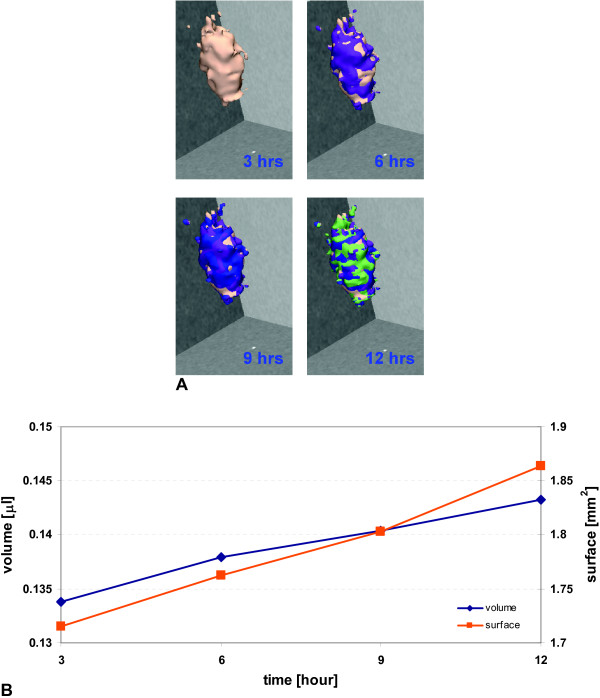
**Volumetric rendering and growth dynamics of the MTS in the collagen I matrix at four consecutive time points**. **(A) **The segmented MTS is reconstructed in 3D and the color-coding indicates the growth increase at each time point. The MTS appears to grow anistropic, which may indicate regional heterogeneities in composition of either MTS or microenvironment, or both, and/or point towards a heterogeneous local interaction between cells and gel. **(B) **MTS volume and surface area are calculated and plotted over time.

**Figure 3:**
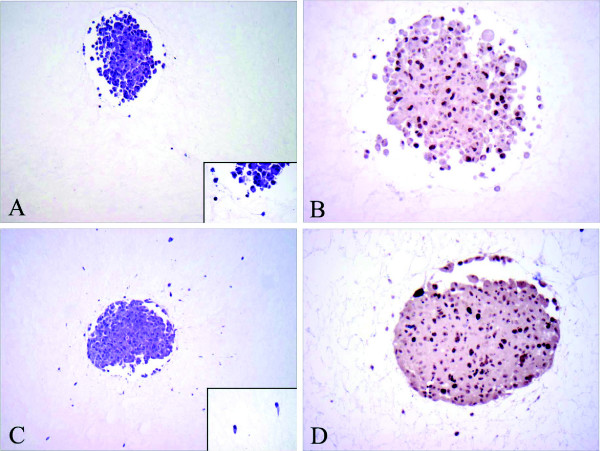
**Histopathology findings, comparing 14T MTS with control**. **(A) **H&E staining of 14T specimen (original magnification ×100) showing only a small number of cells infiltrating the gel. Insert shows infiltrating cells in higher magnification (original magnification ×400). **(B) **MIB-1 immunostaining of 14T specimen (original magnification ×200), highlighting cells that are not in G0 as dark brown nuclei. **(C) **H&E staining of control (original magnification ×100) showing a large number of infiltrating cells in adjacent gel. Insert shows the infiltrating cells in higher magnification (original magnification ×400). **(D) **MIB-1 immunostaining of control (original magnification ×200), highlighting cells that are not in G0 as dark brown nuclei.

The 3D reconstruction shows rather heterogeneous expansion patterns with rough surface growth areas throughout over the course of the observation period (Figure 2(A)). Intriguingly, once started (orange droplets at the MTS' proximal apex at time point '3 hours') glioma cell expansion into the gel seems to give rise to an 'imprinting' process which confirms the 'trailblazer' concept that has been described previously in [2,4]. Finally, corresponding to the MTS' preserved structural viability (compare with Fig. 3) Figure 2(B) also confirms its functionality in that both MTS volume and surface area increase throughout the observation period.

### Histopathology

To semi-quantitatively evaluate the viability of the MTS that has been imaged over 12 hours with 14T MRM, standard immunohistochemistry was performed and the histopathology results were compared with the control (as described above). As documented in Figure 3, the imaged MTS not only remains viable overall but, compared to the control tumors the 14T specimen even contained a substantially larger proliferative fraction (see Table 2). More specifically, the MIB-1 labeling index for the control is 12.5% (30 positive cells out of 239 counted) as compared to the MIB-1 labeling index for the specimen which is with 21.1% (50 positive cells out of 236 cells counted) almost doubled. Furthermore, reviewing comparable sections, in the 14T specimen only 5 cells could be found to invade the gel; conversely, a [total] of 32 cells invaded the collagen matrix in the control experiment. While the min-max values expectedly (given the difference in *n*) showed considerably more heterogeneity in the control group, intriguingly, the overall mean invasion distance was with 2.10 versus 2.11 almost identical between 14T specimen and control. We note that a 2^nd ^control experiment confirmed the results.

**Table 2:** Histopathology.

	**Proliferation**		**Invasion**			
**14T**	50 pos/236	**21.1%**	n: **5**	Mean: **2.10**	SD: 2.07	Min-Max: 0.50–4.70
**Control**	30 pos/239	**12.5%**	n: **32**	Mean: **2.11**	SD: 1.60	Min-Max: 0.30–6.50

To document proliferative activity, we report here the percentage of MIB-1 positive cells for the 14T specimen versus control. The invasion data result from measuring the straight-line distance between an invasive cell and a circle drawn around the spheroid. The measurements are done on a print of a 20× magnification, and represent [arbitrary units]. (See text for more details).

## Discussion

Our data provide clear evidence that magnetic resonance microscopy can be used to study the dynamic growth of multi-cellular tumor spheroid embedded in a collagen I matrix, at a resolution close to the single cell level (Figure 1). Adding 10 mM Gd- DTPA to the gel dramatically reduced the image acquisition time without any detrimental impact on cell viability (Figure 3). Intriguingly, the glioma cells showed distinct spatio-temporal expansion patterns into the gel with spotty surface expansion across the spheroid and the notion of a 'trailblazer' mechanism that guides cell motility in that single cells follow each other along preformed pathways (Figure 2A). The histological results showed surprisingly not only *more *proliferative activity yet concomitantly also *less *cells pursuing invasion in the MRM specimen than in the control. Intriguingly, the mean invasive distance was virtually identical which supports the notion that the phenotype itself remained unaffected. This preliminary data support the notion of a "dichotomy" between proliferation and migration, which was experimentally shown by Giese et al. [19] and modeled with a molecular switching mechanism by Athale et al [20] and Zhang et al [21]. The impact of high field MR (14 Tesla in our study) on cancer cells is somewhat controversial. For instance, Santini et al [22] showed that a sinusoidal 50 Hz magnetic field of 1mT significantly increased spheroid invasive properties, but had no damage on growth. Short et al. [23] showed that for the two cell lines exposed to a 4.7T field up to 72 hours, there was no change in cell growth rate. On the contrary, Raylamn et al [24] demonstrated that human cancer cells exposed to 11.7T for 64 hours had a slower growth rate. In our study, the spheroid also experienced a high frequency small magnetic field (B_1 _field, RF pulse) in addition to the 14T main magnetic field. Although the results from a single case study must be interpreted with proper caution, the increase in proliferation and concomitant reduction in the number of invasive cells observed in our study may indicate that different exposure time and magnetic field strength can impact tumor expansion patterns. Hence, our results warrant a more extended study to investigate in detail the impact of MRM on normal and cancer cell viability and phenotypes.

Kaufman et al [2] showed different patterns of MTS growth and mobility in collagen I matrices of varying concentrations. They observed a more effective invasion in high concentration (1.5 and 2.0 mg/mL) gels at early time. The choice of 1.5 mg/mL concentration used here was a compromise between having a high modulus to maintain sample integrity and allowing cells to move around quickly, and low optical density to allow us to locate the spheroid easily by eye and enable, perhaps, comparisons with optical imaging in the future. Previously, Brandl et al [25] used an 11.7T spectrometer to study multicellular tumor spheroids that were generated from human malignant MV3 melanoma. In their study, the spheroid was allowed to grow for seven to fourteen days in agar to a diameter of 400 – 1000 μm with a starting dense population of 5·10^6 ^cells. This may lead to central necrosis due to limited nutrition and oxygen in the core region. They indeed observed a necrotic core in their sample. The fact that the authors did not observe any morphologic change over the four to five-hour imaging time is less surprising since their agar environment is far more rigid than the collagen I gel used in our study here.

The advantage of using MRM in this context lies in its non-invasiveness, and its true three-dimensional full-scale (global and local) view of MTS' dynamic growth. It is very difficult, if not impossible to acquire whole three-dimension images of MTS in collagen I gel using most modern optical imaging methods. Confocal microscopy allows three-dimensional imaging; however, traditional single photon reflectance or fluorescence methods are limited by the working distance of objectives, typically a few hundred microns. Two-photon confocal microscopy and second harmonic generation can go beyond this limit, but still remain within 1 mm of the surface [26-28]. Conventional light microscopy can surpass this limitation as well, but this is accompanied by a loss in axial resolution. Moreover, a general limitation of light microscopy is that it is constrained by the opacity of tissues. To overcome the loss of signal due to increasing depth, one has two choices – with clear drawbacks: **i) **increasing light power may decrease cell viability very quickly, together with the photobleaching seen in typical fluorescence imaging; **ii) **fixing and staining the cells prior to imaging usually yields a better signal-to-noise ratio than live cells, but the mere fact of fixing the system is an obvious loss in evaluating the time evolution of a living sample. That is not to say of course that optical methods are incapable of generating useful and quantitative information. Interesting studies by e.g. Nygaard et al [29] and recent work by Kaufman et al [2] yield useful information about MTS growth, though with the limitations described above.

With the presently achieved MRM resolution already, we observe "spotty" volumetric changes. Future studies of MTS growth using MRM will reveal even more detailed structural changes in a true 3D sense as the image resolution further improves. MRM is beginning to bridge the imaging scale that, until recently, has been accessible to optical techniques only, with the clinical MRI data level which is still plagued by a rather poor spatial and temporal resolution. Such three-dimensional volumetric studies of the morphologic growth of MTS using MRM will provide complementary information about the growing tumor and the dynamic interaction with its microenvironment. With the help of integrative *in silico *modeling insights gained with MRM *in vitro *can help us understand better the complex cancer growth patterns seen in patients.

## Conclusion

We have shown that magnetic resonance microscopy can be used to study dynamic tumor growth *in vitro *with a resolution that approaches that of light microscopy. Based on this microscopic MRI pilot data, we argue that one may be able to use this clinically relevant imaging modality also *in vitro*, particularly once combined with molecular imaging techniques. Being able to rely on *one *imaging modality only, or even primarily, would greatly enhance our ability to successfully integrate *in vitro *with *in vivo *data, using innovative *in silico *models that bridge between these scales.

## Competing interests

The author(s) declare that they have no competing interests.

## Authors' contributions

SH carried out the MRM studies and participated in the image analysis. GD was responsible for the MRI pulse sequence development. DV was responsible for sample preparation and cell culture, whereas ZW carried out the 3D image reconstruction and calculated the growth dynamics. ASR performed and interpreted the histopathological and immunohistochemical analyses. BRR and DAW helped direct the MR imaging and materials science aspects involved. TSD conceived the study, led its overall design and coordination, and helped to draft the manuscript. All authors read and approved the final manuscript.

## Pre-publication history

The pre-publication history for this paper can be accessed here:


